# Accelerating access to diagnostic tools: perspectives from the joint international tropical medicine meeting 2023

**DOI:** 10.1093/trstmh/traf022

**Published:** 2025-03-06

**Authors:** Puttarin Kulchaitanaroaj, Philip J Turner, Cheikh T Diagne, Maytouch Lojanarungsiri, Maneerat Ekkapongpisit, Rosanna Ŵ Peeling

**Affiliations:** Mathematical and Economic Modelling Department, Mahidol-Oxford Tropical Medicine Research Unit, Mahidol University, Bangkok 10400, Thailand; Nuffield Department of Primary Care Health Sciences, University of Oxford, Oxford OX2 6GG, UK; DIATROPIX, Institut Pasteur de Dakar, Dakar BP 220, Senegal; Mathematical and Economic Modelling Department, Mahidol-Oxford Tropical Medicine Research Unit, Mahidol University, Bangkok 10400, Thailand; Mathematical and Economic Modelling Department, Mahidol-Oxford Tropical Medicine Research Unit, Mahidol University, Bangkok 10400, Thailand; London School of Hygiene and Tropical Medicine, London WC1E 7HT, UK

## Letter to the Editor

Despite an ever-expanding portfolio of diagnostic tests and an unprecedented surge in test research and development during the severe acute respiratory syndrome coronavirus 2 (SARS-CoV-2) pandemic, timely access to quality-assured diagnostic tests remains a major challenge. This article offers suggestions on accelerating diagnostic access at the policy level, encouraging the use of diagnostics in primary care settings and routes to establish an ecosystem of diagnostic development based on the preconference workshop for global diagnostic tools development for tropical diseases at the Joint International Tropical Medicine Meeting in 2023 and subsequent communications.

It takes approximately 10 years for diagnostics to pass regulatory approval and be adopted.^1,2^ The process includes analytical evaluation and field trials, regulatory review and approval, policy development through health technology assessment, guideline development and test adoption. Globally, such pathways are quite fragmented with unnecessary duplication and considerable barriers. A project called the Accelerating Diagnostic Access Project was initiated to identify possible ways to accelerate diagnostic access. The project launched capacity building and simulation exercise workshops with regulatory bodies, public health officials, and scientific key opinion leaders in Africa, Latin America and Asia, as well as with the diagnostics industry, to summarize key messages (Supplement 1) and consolidate the framework for acceleration of diagnostics access. Accelerating diagnostic access will be critical for dealing with antimicrobial resistance and epidemic preparedness. Key messages from different regions and the industry include the following:

1. Tools to accelerate access, such as Emergency Use Authorization, regulatory reliance, convergence and harmonization, exist, but uptake is slow.
2. There is a need for mechanisms to prevent unnecessary duplication of clinical performance studies, such as diagnostic evaluation networks, joint review of data by regulators and policy makers and data sharing across countries.
3. There is a need for capacity building for regulators and policymakers on advances in diagnostic technologies, data digitization and connectivity for surveillance.
4. There should be advocacy on understanding the true value of diagnostics and effective communication to all stakeholders including the public sector.

Key settings that may benefit from the acceleration policy-related plan for accessing diagnostic tools are primary and community healthcare settings. The current diagnostic-testing model from primary and community care usually requires specimen transportation with associated delays and the potential for the development of transport-related artefacts such as loss of microorganism viability prior to laboratory culture. With appropriate use of point-of-care In Vitro Diagnostics (IVDs) and medical technologies in those settings, early detection and management of disease can reduce unnecessary referrals, improve antibiotic prescribing, prevent disease progression and lower healthcare costs. However, there is little exploration of the demand and input from the community in the development of new diagnostic tools. Moreover, barriers to implementation of IVDs in primary care include a paucity of real-world performance, and clinical and cost-effectiveness estimates for community use cases for technologies.

A challenge for healthcare commissioners contemplating the introduction of new diagnostic tests are mismatches between test performance estimates published in approved documentation for regulatory purposes (e.g. the manufacturers’ Instruction For Use [IFU] documents) and the performance of diagnostics in independent studies. This phenomenon has been termed ‘optimism bias’^3^ and was clearly illustrated in rapid antigen tests for SARS-CoV-2 in a review that found 12/22 IFUs of rapid tests had statistically significant higher estimates of test sensitivity than estimates from a Cochrane review of independent studies.^4^ Optimism bias has potentially been driven historically by regulations that have lacked requirements for performance evaluations to incorporate real-world estimates or checks. One of the solutions is that manufacturers, researchers and government agencies should ensure diagnostic performance, not only from the technical perspective, but also the clinical performance by performing tests in populations and settings where those tests will be used.^5,6^ Also, the performance capacity of the diagnostic tools needs to be maintained in the entirety of their life-time use.^7^

**Figure 1.**
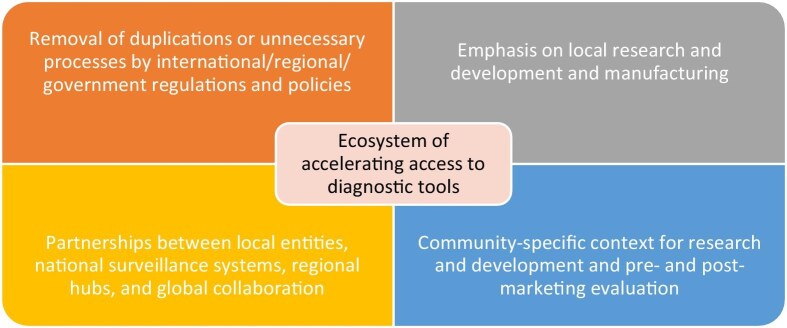
Summary of the ecosystem of accelerating access to diagnostic tests.

Regarding the development and commercialization of diagnostics, aspirational targets to improve access and implementation success will include tests that can be done at the point of care, with minimal sample preprocessing, faster detection time, improved performance (i.e. sensitive and specific) and at lower cost. To achieve this, government regulations/policies and partnerships between sectors can alleviate problems. For instance, policies promoting local development of IVDs and reducing imported IVDs, supporting the formation of partnerships (e.g. university–local industry collaboration) and aiding the growth of local biotechnology companies will help to create an ecosystem of IVD development for sustainability. An example of such an ecosystem led by the Institut Pasteur de Dakar in Senegal is the Integrated Disease Surveillance, which incorporates several components of a genomic platform, innovation development, capacity building, syndromic surveillance at different sites in the country, environmental consideration, listening to communities and using a one-health model for evaluation. Essentially, diagnostics should be considered added value through their ability to avert costs or complications. To accelerate access to diagnostic tools, the components of an efficient ecosystem have been proposed (Figure 1).

## Supplementary Material

traf022_Supplemental_File

## Data Availability

The data underlying this article are available in the article and in its online supplementary material.
