# Supplementary material for: Accelerating access to diagnostic tools: perspectives from the joint international tropical medicine meeting 2023
Source: Trans R Soc Trop Med Hyg. 2025 Mar 6;119(10):e10–2. doi: 10.1093/trstmh/traf022 (PMC12486380; doi:10.1093/trstmh/traf022)
Supplement: traf022_Supplemental_File [file traf022_supplemental_file.docx]

**Supplement: Accelerating access to diagnostic tools: perspectives from the joint International tropical medicine meeting 2023**

Table 1 Key opinion from the region and industry on accelerating access to quality-assured diagnostics

| Topic/Region | African region | Latin America region | Asian | Industry |
| --- | --- | --- | --- | --- |
| About patient benefits |  |  |  |  |
|  | Saving lives is a priority over costs | There should be promotion of clear discussions on the risks, benefits, and roles of  early introduction of new diagnostic tools. |  | Diagnostics are often undervalued. The government focuses on added costs rather than added value to the healthcare system. It is important to consider the value to society and cost averted by preventing misdiagnosis, severe disease due to late diagnosis and use of wrong therapeutics. |
| Quality |  |  |  |  |
|  | There should be no compromise on quality or evidence. | There should be standards for a regulatory purpose such as use of approved standards that can be applied to medical devices and IVDs, shortening the pathway—ISO 13485 or other Regulators’ Good Manufacturing Practice (GMP) Certificates.  All different types of stakeholders should have roles in promoting the quality standards, including an active participation through Assessment Programs that: a) give performance indicators to the market and b) work as a post market surveillance tool for regulators. | There should be laboratories with good quality and evaluation with qualified biological samples. | Barriers and challenges in accessing biological materials impede test development, early-stage evaluation and quality assurance. |
| Networking |  |  |  |  |
|  | There should be a framework for building regulatory capacity for diagnostics (including reliance and convergence), regional and sub-regional hubs, a common platform to share knowledge and best practice, multi-center studies for licensing, trust among all stakeholders, ways to overcome culture and language differences, community involvement, flexible regulation, and clear definition of roles and responsibilities. | Regional/global networks supporting each other are crucial.  Reliance as one of the key regulatory tools for accelerating access to innovative diagnostics. Other tools to expedite regulatory approvals: EUA—Emergency Use Authorization, Medical Device Single Audit Program (MDSAP), and Post Market Surveillance Reports.  There should be sharing clinical trials and studies, as well as sharing cross-border public health programs.  Developing a common ground for sharing all the achievements and knowledge, like the Inter-American Coalition for Regulatory Convergence, PAHO, ALADDIV, IDC, among other institutions is important. | Regulatory should be a focus. There should be a regional platform for diagnostic approval, diagnostics development standards, and ethical framework to guide diagnosis development in ASEAN.  Regional pre-market product validation and post-market surveillance mechanism also should be developed.  There are suggestions on developing a network of lab-of-excellence across ASEAN, shared databases for diagnostics use cases and their performance, and strengthening supply chain of reagents and materials for product development and manufacturing.  There should be a global platform for global discussions on diagnostics e.g. World Health Assembly for Diagnostics. This would require cooperation by scientific academics and ministries to initiate the process. | A framework or mechanism should be developed to harmonize and standardize the determination of the value of diagnostics.  Duplication of clinical performance studies delays potentially life-saving diagnosis and increases costs for companies, driving up product prices.  Advocacy and effective communication need to continue throughout the pandemic as well as post pandemic |
